# Bird Feces as Indicators of Metal Pollution: Pitfalls and Solutions

**DOI:** 10.3390/toxics8040124

**Published:** 2020-12-18

**Authors:** Tapio Eeva, Nelli Raivikko, Silvia Espín, Pablo Sánchez-Virosta, Suvi Ruuskanen, Jouni Sorvari, Miia Rainio

**Affiliations:** 1 Department of Biology, University of Turku, 20014 Turku, Finland; skruus@utu.fi (S.R.); jousor@utu.fi (J.S.); miikoi@utu.fi (M.R.); 2 Department of Environmental and Biological Sciences, University of Eastern Finland, 70211 Kuopio, Finland; nelli.raivikko@gmail.com; 3 Area of Toxicology, Department of Socio-Sanitary Sciences, University of Murcia, 30100 Murcia, Spain; silvia.espin@um.es (S.E.); pablo.s.v@um.es (P.S.-V.)

**Keywords:** bird feces, calcium, heavy metals, measurement error, pied flycatcher, repeatability, uric acid

## Abstract

Bird feces are commonly used as a proxy for measuring dietary metal exposure levels in wild populations. Our study aims to improve the reliability and repeatability of fecal metal measurements and gives some recommendations for sampling. First, we studied levels of variation in metallic element (arsenic, calcium, cadmium, cobalt, copper, nickel, lead) concentrations: temporal variation within an individual, among siblings in a brood and among-brood/spatial variation. Second, we explored the variation caused by dual composition (urate vs. feces) of bird droppings. Two sets of fresh fecal samples were collected from pied flycatcher (*Ficedula hypoleuca*) nestlings living in a metal polluted area in summers 2017 (dataset 1) and 2018 (dataset 2). We found a great deal of temporal intra-individual variation in metal levels, suggesting that dietary exposure varied markedly in a short time scale (within a day). A sample from only one nestling per brood did not well describe the brood mean value, and we recommend that at least four siblings should be sampled. Brood level samples give relatively good temporal repeatability for most metals. For all the metals, the levels in the fecal portion were more than double to those in the urate portion. Since the mass proportion of urate in the bird droppings varied a great deal among samples, standardizing sampling, e.g., by collecting only the fecal part, would markedly reduce the variation due to composition. Alternatively, urate portion could be used for biomonitoring of internally circulated bioavailable metal.

## 1. Introduction

Birds are widely used as biomonitors to give information on the state of the environment—for example, the levels of environmental pollution [1,2,3,4]. In this respect, non-destructive monitoring methods are urgently needed for ecotoxicological field studies, which sometimes include also endangered species [5,6,7]. Bird feces are often used as a proxy for measuring dietary contaminant exposure levels [8,9,10,11]. For example, the levels of non-essential metals in the feces of passerine birds reflect well the pollution loads in the environment and correlate with the levels measured in their food items, and this methodology is therefore widely used [12,13]. Collecting feces from nestlings is relatively easy and can be done, e.g., by voluntary bird ringers, since nestlings often defecate when they are handled for marking and measuring. In addition to measuring pollutants, bird feces are useful for measuring diet with DNA meta-barcoding, some physiological biomarkers such as stress hormone levels and gut microbiome [14,15,16,17,18]. At best, all this information can be obtained from the same sample. On the other hand, it is not always clear what can be concluded on the fecal pollutant levels [19], and there are several potential methodological pitfalls, such as unknown temporal and spatial variation in concentrations. For example, it is not clear how well a sample collected in a single time-point reflects the general exposure level of an individual.

Birds are exposed to metals via food, water, inhalation and preening their feathers, but food is in most cases considered the most important route in wildlife [20,21,22]. In general, metals are relatively easily absorbed as soluble salts, but the proportion absorbed from food varies, e.g., among metals and their form, bioavailability, bird species and food quality [23,24,25]. For example, fecal concentrations of essential elements like copper (Cu) and zinc (Zn) tend to show weaker correlations with internal levels than some of the more toxic non-essential elements, such as arsenic (As) or lead (Pb) [19,26]. This is probably because animals can better regulate the absorption of essential mineral elements, although this may fail as well at very high exposure levels [27,28]. The unabsorbed part will be excreted together with the feces. However, bird droppings consist of two components: feces and urine, the largest component in the latter being derivatives of uric acid or its salts (urate) [5,29,30]. Uric acid contains nitrogenous waste but it is also a route for excreting metals from the bloodstream, initially absorbed from the gut [5,31]. Therefore, when bird droppings are measured for their metal content, it remains unclear how much the concentration reflects the unabsorbed part in feces and how much the internal route through kidneys to uric acid and through bile to feces [32].

The aim of our study is to improve the reliability and repeatability of fecal metal measurements. For this, we have collected two sets of fecal samples from nestlings of an insectivorous passerine, the pied flycatcher (*Ficedula hypoleuca*), to explore the sources of variation in metal levels. First, we want to reveal the most important levels of variation by variance partitioning: temporal variation within individual, among siblings in a brood and among-brood/spatial variation. Second, we want to explore the variation caused by dual composition (urate vs. feces) of bird droppings. Most studies using bird droppings for biomonitoring of environmental metal levels have used whole feces, e.g., [1,11,33,34]. However, this is a potential source of measurement error, because the proportions of feces and urate vary in samples, and information on their relative metal concentrations is lacking. Information on metal exposure levels is often combined with some physiological or fitness-related variables. If there are unknown sources of variation in sample concentrations, finding a connection between the exposure level and response may fail. Finally, we want to give some practical recommendations for improving sampling and the reliability of results. Reliability of laboratory analyses, differences in diet quality and absorption and connections between fecal concentrations and internal tissue levels are, however, beyond the scope of our study.

## 2. Materials and Methods

### 2.1. Study Area and Study Species

The data were collected in summers 2017 and 2018 around a copper-nickel (Cu-Ni) smelter (61°20′ N, 22°10′ E) in Harjavalta town, Southwestern Finland. Sulphur oxides (SO_x_) and metallic elements (especially As, cadmium Cd, cobalt Co, Cu, Ni, Pb) are common pollutants in this area [35,36,37]. Elevated metal concentrations occur in the soil, vegetation and fauna of the polluted area due to current and historical (since 1945) deposition [35]. More details on emissions and metal levels in birds are given in Eeva et al. [38] and Berglund et al. [11,39]. Six study plots with nest boxes [40] were established in different directions < 2 km from the smelter (Supplementary Materials, Table S1).

A small migratory and insectivorous bird, *F. hypoleuca*, is the most abundant species breeding in our nest boxes and its population has been studied in this area since 1991 [41]. The diet of nestlings consists of wide variety of insects and other arthropods, which parents collect relatively close to their nest [42]. Therefore, the dietary metal exposure of nestlings reflects the concentrations in local invertebrate fauna and local contamination in general. An earlier investigation in the same study area indicated that the metal levels in the feces of *F. hypoleuca* nestlings showed strong positive correlations to the metal levels in soil, vegetation and insects [37]. Both parents feed the nestlings, and the average sized brood (circa 6 nestlings) receives on average 32 food loads per hour [43]. Mean (±SD) brood sizes in our sample broods in 2017 and 2018 were as follows: 5.5 ± 0.80 and 5.9 ± 1.07, respectively.

### 2.2. Sampling

In summer 2017, we collected 396 fecal samples from 208 *F. hypoleuca* nestlings in 38 broods at six study plots (dataset 1; Supplementary Materials, Table S1). Each brood was sampled twice during the same day (and only in one day) to get paired samples from the same nestlings, the mean (±SD) interval between the two successive samples being 5.6 ± 1.3 h. Nestlings were 5–12 days (mean ±SD: 8.4 ± 1.8) old. The sampling period to obtain samples from all broods took 10 days in 2017.

Fresh fecal samples were taken individually into clean 1.5 mL polypropylene tubes when nestlings were individually marked with aluminum rings (licenses by the Finnish Center for Economic Development, Transport and the Environment: VARELY/959/07.01/2012; date of approval: 7 Jan 2012; VARELY/3622/2017; date of approval: 11 Dec 2017). Nestlings of this species normally defecate during ringing but this can also be induced by pressing gently the cloaca, e.g., with a round-ended hairpin. Samples were frozen at −20 °C, dried later in the laboratory at 40 °C for 72 h and ground to powder. Both fresh and dry mass was taken to estimate the water content (mean ± SD) of samples, which was 79.0 ± 6.1%. Dry to wet conversion factor for metal concentrations was then 0.210.

In summer 2018, we collected 40 fecal samples from 7–11-day-old nestlings from six study plots and 20 broods, always sampling two randomly chosen nestlings in a brood, but this time only one sample per nestling was taken (dataset 2; Supplementary Materials, Table S1). The sampling period to obtain samples from all broods took 6 days in 2018. Sampling itself was otherwise similar to the previous study, except that we now split each sample into two parts: fecal part (dark feces) and urate (white solid urine). This was done on a clean plate by carefully peeling the white urate from the top of the feces with a plastic teaspoon. However, small amounts of urate may have remained within the feces and vice versa. Samples were then treated as described above. Water contents (mean ± SD) of the fecal and urate parts were 76.8 ± 8.9% and 73.3 ± 8.9%, respectively. Dry to wet conversion factors for metal concentrations were then 0.232 for the feces and 0.267 for the urate.

### 2.3. Trace Element Analyses

For 2017 samples, element concentrations (aluminum Al, As, boron B, Cd, calcium Ca, chromium Cr, Co, Cu, iron Fe, Pb, lithium Li, magnesium Mg, manganese Mn, molybdenium Mo, Ni, phosphorus P, potassium K, rubidium Rb, silicon Si, sodium Na, strontium Sr, sulphur S, titanium Ti, Zn) were determined in University of Murcia with an inductively coupled plasma optical emission spectrometer (ICP-OES) Thermo Scientific iCAP 6500 Duo (Thermo Fisher Scientific Inc., Waltham, MA, USA). Dried samples (*n* = 396) were placed in digestion tubes to which a mixture of 4 mL HNO_3_ (70%) and 1 mL H_2_O_2_ (33%) was added. The sample was then submitted to progressive thermal treatment and, after the microwave procedure, the sample was diluted in ultrapure water before analysis. The limits of quantification (LOQs) ranged between 0.01 and 1 μg/g, and the accuracy of measurements was in the range of 94−108%, with uncertainties below 8% in all cases. Five samples, typically with small sample mass (<0.01 g) and deviating concentrations for several elements (>20 times higher or lower than the mean value), were discarded from all the subsequent analyses, the final sample size being 391.

For 2018 samples, element concentrations (As, Ca, Cd, Co, Cu, Ni, Pb) were determined in University of Eastern Finland with an inductively coupled plasma mass spectrometer (ICP-MS) PerkinElmer NexIon 350D-AMS (PerkinElmer Inc., MA, USA). Dried samples (*n* = 80) were placed in digestion tubes with 8 mL HNO_3_ (67−69%). The sample was then submitted to progressive thermal treatment and, after the microwave procedure, the sample was diluted in ultrapure water before analysis. Calcium levels were too high to give reliable results, and they are not reported. Samples of Bovine Liver 1577b (for Cd, Cu and Pb) and Tomato Leaves 1573a (for Co, Ni and As) were used as certified reference materials (SRM; Sigma-Aldrich, St. Louis, MO, USA). Recoveries (%) were as follows: 97.6% for As, 71.6% for Cd, 86.6% for Co, 92.5% for Cu, 102% for Ni, 192% for Pb. Since observed Pb values were high as compared to the certified values, the absolute levels of this metal should be interpreted with caution. All concentrations are reported on a dry mass basis (d.m.). For urate, one sample was discarded from all the subsequent analyses because of exceptionally low (>100 times lower than the mean value) concentrations for most elements. Furthermore, one exceptionally high value for Cu (16 times higher than the mean) was omitted.

### 2.4. Statistics

All the analyses were run with statistical software SAS 9.4 [44]. For all 24 elements in dataset 1 (2017), we first calculated ranges and quartiles and checked the proportions of values below the limit of quantification (<LOQ) to assess data quality (Supplementary Materials, Table S2). Because, in all cases, the proportion of <LOQ values was less than 15%, we substituted them by a random number between 0 and LOQ [45]. There were no <LOQ values in dataset 2 (2018). Associations among elements in dataset 1 were further inspected with a hierarchical cluster analysis [44] by using Pearson correlation matrix with average linkage method for clustering (Supplementary Materials, Figure S1). With this exploratory analysis, we sought to determine which elements showed similar distributions among the samples, indicating similar spatial distribution and source.

Six main pollutants (As, Cd, Co, Cu, Ni and Pb) of the smelter, together with Ca, were selected for further analyses. Selection of toxic elements was based on earlier information on the emission profile of the smelter [35,37]. Calcium was included because it interacts with toxic metals, and therefore it is often measured together with the other metals [46,47]. These concentrations were first log_10_ transformed to make them better conform to normal distribution, which was confirmed by visual inspection of histograms. Because the age of the broods varied, we first assessed whether metal concentrations were age-dependent by using a linear regression model. All metals, except Cd, showed a decreasing trend with age (result not shown), and therefore we used age-corrected residuals in all the following analyses. This was important in order to avoid mixing the age effect with the other sources of among-brood variation.

The differences in metal levels among plots, broods, nestlings and sampling times were inspected with general linear models (GLM procedure in SAS) with fully nested structure (i.e., brood nested within plot and nestling nested within brood; Table 1). In such models, also mean squares need to be compared sequentially, i.e., the correct denominator in the test for the first factor is the mean square due to the second factor, and so on [44]. Model estimates (least squares means and confidence limits) for log_10_-transformed values were back-transformed to the original scale for tables. After this, we partitioned the variation by using a similar hierarchical model in the NESTED procedure of SAS to inspect which levels in our hierarchical data showed the most variation (i.e., among study plots, among broods within a plot, among nestlings in a brood and between two sampling occasions for each nestling within a day; Table 2) [44]. We also calculated Pearson correlation coefficients and repeatabilities between the metal levels in successive samples (Table 3). Repeatability values (intra-class correlation coefficients) were calculated following Lessells and Boag [48] by including only those cases where we had a value from both successive sampling occasions. Repeatabilities are given for individual, brood (average value of nestlings of a brood) and plot (average value of all broods within a study plot) levels.

Finally, we explored for each metal how many nestlings within a brood need to be sampled to obtain a representative value for a brood mean. For this, we calculated Pearson correlation coefficients between the brood mean element concentrations and the mean concentrations for increasing numbers of randomly chosen nestlings within brood. From all the brood-level samples (two successive samples from 38 broods = 76), we only included for this comparison those with at least five measurements (i.e., at least five nestlings sampled), leaving 59 brood-level samples for the analysis.

In the 2018 data, where the fecal part and urate were separately weighted and measured for their metallic element concentrations, the fecal part represented on average 61% (range 23−94%) of the total (i.e., feces and urate combined) sample mass. We calculated how large a proportion (%) of the total metal content in a combined sample was included in the fecal part, by taking into account the concentrations and relative masses of the two components. Thereafter, we ran for each metallic element a linear regression model where the mass proportion (%) of the fecal part was used to explain the standardized (mea*n* = 0, SD = 1) metal concentrations of the combined sample (Table 4). This will indicate how sensitive the total concentration is to the variation in mass proportions of feces and urate. The significance level was set at *p* < 0.05 in all analyses.

## 3. Results

### 3.1. Levels of Variation (Dataset 1): Among Plots, among Broods, within Broods and within Individuals

Metal levels showed significant spatial variation: among-brood variation was significant for all metals and the variation among study plots was significant for Ca, Ni and Pb (Table 1). Within-brood variation was significant for Co, Cu, Ni and Pb (Table 1). However, by far, most of the variation (on average 66%) in metal levels took place in time (i.e., between two successive samples from the same nestling within a day), indicating that there is a great deal of short-term temporal variation and especially so for the levels of Ca and Cd (Table 2). The second largest share of variation took place among broods, although for Cu and Pb, within-brood variation was slightly higher (Table 2). The least variation took place among the study plots (Table 2), suggesting relatively even pollution load over the whole area. Although our partition of variation suggested that a relatively small proportion of variation took place within brood (Table 2), this was mainly because there were always two samples per nestlings. If two sampling occasions were analyzed separately, within-brood variation would be the largest source of variation for all elements (e.g., in Ca explaining circa 85% of variation in both sampling occasions).

**Figure 1:**
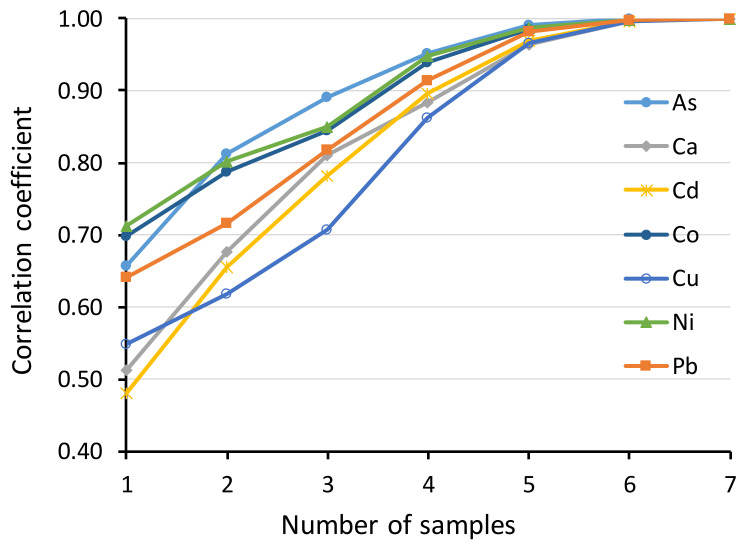
Pearson correlation coefficients between the brood mean element concentrations and the mean concentrations for increasing numbers of randomly chosen nestlings within brood. *N* = 59.

Because of the large temporal within-individual variation, Pearson correlation coefficients between the first and second samples were relatively low (all <0.4), although significant for all metals, except Ca (Table 3). At individual level, repeatability was low (<0.4, see [49]) for As, Ca, Cd, Cu and moderate (0.4−0.7) for Co, Ni, Pb (Table 3). Calcium showed a value close to zero, which means that the repeatability is not essentially better than between random values. Brood-level repeatability was still low for Ca and Cd, moderate for As and high (>0.7) for the other metals (Table 3). At plot level, repeatability was still low for Cd, moderate for Ca and Cu and high for the other metals (Table 3). Pearson correlations between the brood mean element concentrations and the mean concentrations for increasing numbers of randomly chosen nestlings indicate that sampling at least four nestlings would produce a good correlation (r > 0.85) with brood mean for all the studied elements (Figure 1).

### 3.2. Levels in Feces and Urate (Dataset 2)

On average, 61% of the sample dry mass consisted of feces, the rest being urate (Table 4). For all the metals, the levels in the fecal portion were more than double (on average 2.2 times higher) those in the urate portion (Table 4). Since the water content was relatively similar in the two matrices, this ratio would not be very different in fresh samples (1.8−2.2 times higher in feces). When taking into account the relative mass of the two components, circa 75% of the total metal load is stored in the fecal part, the proportion being relatively constant among the six studied elements (Table 4). In the regression models, variation in the mass proportion of fecal part explained 0.0–9.3% of the variation in total metal concentration, depending on the element and showing highest value for As (Table 4). Concentrations correlated positively and significantly between the two matrices for all elements (Table 4).

## 4. Discussion

Significant among-brood variation for all metals suggests that fecal levels are a useful measure of metal pollution exposure at brood level (i.e., they indicate spatial differences), although the repeatabilities for Ca and Cd remained relatively low. As a rule of thumb for this species, we recommend sampling at least four nestlings, since this would produce a good correlation with brood mean for all the studied elements (Figure 1). Samples can be pooled by brood or analyzed individually to retain the information on within-brood variation, but the latter alternative would cause much greater analytical costs. An alternative, but more time-consuming, option would be sampling the same individual twice (or more) and combining the samples for metal analysis. This would level off most of the short-term temporal variation. Especially for Ca, it might be reasonable to use the average of several nests within a study plot to describe general levels in the area. Due to large within-individual variation, using metal levels of a single individual fecal sample, e.g., for explaining some physiological or fitness parameters, cannot be recommended, although even this may work if the range of metal exposure levels is large.

Relatively large short-term temporal variation in fecal metal levels likely arises from varying dietary invertebrate composition. Food retention times in nestlings of small insectivorous birds are short (circa 1 h), and a single item of bird dropping may include remains of several consecutive meals [50,51]. *F. hypoleuca*, like many other insectivorous passerines, feeds nestlings with a high diversity of invertebrates [42] and successive food loads contain varying species with different metal composition [52]. This is especially the case with the essential element Ca, since birds need to search for specific Ca-rich food items (e.g., snails, millipeds and woodlice) to fulfill the Ca requirements of growing nestlings [53,54]. Therefore, some food loads contain such Ca-rich invertebrates while some do not. For example, in another insectivorous passerine, the tree swallow (*Tachycineta bicolor*), 33% of nestling stomachs contained specifically Ca-rich food items [55]. This would manifest in high temporal or within-brood variation in fecal Ca concentrations. It is also possible that specific Ca-rich food items are found farther from the nest than other types of food, which could mean that several pairs breeding in the same study plot exploit some common Ca sources [56]. This might explain the relatively low among-brood variation in Ca levels (Table 2). The rich Ca content of diet is known to mitigate the harmful effects of toxic metals and, on the other hand, toxic metals can alter Ca metabolism, e.g., by interfering with its transport and storage or substituting Ca at important Ca-binding sites [24,46,57,58].

Another metal showing large temporal variation was Cd, which may again reflect uneven accumulation of this metal among invertebrate species. Cadmium shows high mobility in the soil–plant system and tends to accumulate, e.g., in wood ant species (*Formica* sp.), which use phloem sap excreted by aphids (Aphidoidea) as their main food [59]. Wood ants are an important food source for *F. hypoleuca* [43,60] and, together with relatively low Cd levels in our study area, the varying amounts of ants in the diet could explain the high temporal variation in fecal concentrations of this metal. Likewise, relatively high within-brood variation in fecal Cu levels (Figure 1) could reflect the proportion of spiders (Araneae) in the diet [52]. Spiders are an important prey group for *F. hypoleuca* and typically contain more Cu than insects because they use Cu containing hemocyanin in their hemolymph as an oxygen carrier protein [43,61]. Therefore, the Cu content of a fecal sac may vary according to how many spiders happened to be recently fed to a chick. Instead, the availability of As, Co and Ni showed less within-brood variation (Figure 1), which may indicate that these smelter emitter metals are more evenly available from different food items. These three metals further showed the highest correlations between feces and urate (Table 4), which suggests that their fecal levels correlate better with internal levels than those of the other metals, as was earlier documented for As [26]. In general, short-term variation in dietary levels may explain why some studies have found poor correlations between the fecal and dietary levels [62]. Decreasing trends in metal levels during nestling growth might arise from changing dietary composition or decreased digestive efficiency during nestling growth [48]. The sex of the sampled individuals was unknown but a recent study indicated that both internal and fecal metal levels of nestlings are independent of sex in this species [19].

Differences among plots likely reflect their varying direction and distance from the main pollution source (smelter; Table S1) and spatial location of different functions (e.g., foundry, tailings) within the industrial area. A common emission source was, however, evident in the clustering analysis (Figure S1) for some of the main pollutants of the smelter (Pb, Co, Ni, As). Elements also likely grouped together according to their similar dietary availability, biological activity, homeostatic control (e.g., Cu + Mn) and chemical or geological properties (e.g., Ca + Sr, Cd + Zn, P + S, Mg + K, Fe + Cr + Ti). For example, alkaline earth metals Ca and Sr typically occur together in biological samples because of their similar pathways of uptake and utilization [63,64]. Likewise, Cd and Zn show similar chemical properties, with Cd showing high affinity in Zn binding sites in biological systems [65].

We found a marked difference in metal concentrations between urate and fecal components, the latter showing around two times higher levels. Since the mass proportions of feces in the bird droppings varied significantly (from 23 to 94%) among samples, standardizing sampling, e.g., by collecting only the fecal part, would markedly reduce the variation in metal levels due to composition. For example, the total concentration of As would increase 1.7 fold over this range just by changing the composition. This may partly explain the large variation among individual samples, which was evident in the first part of our study and again warns against using single fecal measurements, e.g., for explaining individual fitness measures. Despite this, standardizing sampling, e.g., by collecting only the fecal part, would reduce the variation in the level of whole data due to the composition being only less than 10% for all metals (Table 4). Therefore, even though error variation due to composition can be high among individual samples, it was, on average, relatively low.

The urate portion could be used as a biomonitor of internally circulated bioavailable metal, which is transferred via blood and kidneys. We would expect this measure to be better associated with any fitness variable than fecal measures, which partly reflect the unabsorbed metal fraction. Significant correlations between metal levels of feces and urate, however, indicate that also feces do reflect internal burden, although there is independent variation as well. This may relate to the fact that part of the metals found in feces also comes through an internal route via liver into bile [66]. For example, in rats (*Rattus norvegicus*), the biliary route is known to have an important role in the excretion of metals like As, Cd, Cu and Pb [67]. The roles of urinary and biliary routes are likely species and metal-dependent, and, e.g., for As in mammals, depend on the chemical speciation [68]. However, we are not aware of any study which has attempted to measure directly the proportional importance of urinary and biliary metal secretion in birds.

In the first part of our study, we partitioned variation in metal levels by using whole droppings, including feces and urate. However, analyzing sources of variation separately for the two components would be interesting too. Metals in urate are expected to be primarily of internal origin, and avian half-life of, e.g., Pb in blood and soft tissues is estimated to range from weeks to months [69]. Therefore, we would expect that urate metal levels show smaller within-individual variation in a short time scale due to varying diet. If so, measuring exposure levels from urate samples could decrease unwanted noise in the data. For measuring feces and urate separately, we would need a simple way of separating urate from feces in relatively small samples. Clapp et al. [5] report one method for separating urate spheres from feces by using 200 g samples of chicken guano, but we do not know if this would be successful for much smaller samples of small-sized insectivorous passerines (dry mass of a single *F. hypoleuca* dropping was 55 ± 30.6 mg).

## 5. Conclusions

Due to considerable among-individual and short-term within-individual variation in the metal levels of bird droppings, we recommend sampling at least four nestlings in a brood to obtain a representative sample describing the brood level exposure. Using metal levels of a single individual fecal sample, e.g., for explaining some physiological or fitness parameters, cannot be recommended. However, these recommendations may not be directly applicable to other species or environments, e.g., because of different diets and physiology. Due to marked differences in metal levels and mass proportions between urate and feces, standardizing sampling, e.g., by collecting only the fecal part, would markedly reduce the variation due to composition. Alternatively, urate portion could be used as a biomonitor of internally circulated bioavailable metals. Information on levels of variation will help in planning the sampling schemes for obtaining representative measurements, and we encourage for conducting similar comparisons in other species and tissues (e.g., blood).

## Figures and Tables

**Table 1:** Geometric means (±95% confidence limits) and hierarchical linear models ^a^ for element concentrations (μg/g, d.m.) in feces of the pied flycatcher (*Ficedula hypoleuca*) nestlings. *N* = 391 samples.

Mean (CL)		As		Ca		Cd		Co		Cu		Ni		Pb	
		4.08 (3.48–4.80)		1932 (1652–2258)		5.51 (5.10–5.95)		3.19 (2.92–3.49)		248 (229–265)		50.4 (46.2–54.8)		5.83 (5.31–6.41)	
	*df*	F	*p*	F	*p*	F	*p*	F	*p*	F	*p*	F	*p*	F	*p*
Plot	5	1.81	0.14	2.92	0.028	0.54	0.75	2.42	0.057	1.75	0.15	3.39	0.014	3.09	0.022
Brood (plot)	32	5.11	<0.0001	1.78	0.011	2.44	0.0001	4.24	<0.0001	2.61	<0.0001	4.25	<0.0001	2.42	0.0002
Nestling (brood)	170	1.17	0.15	0.80	0.93	1.16	0.16	1.68	0.0003	1.56	0.0016	1.53	0.0024	1.53	0.0024

^a^ Linear models with nested structure for age-corrected values (i.e., residuals from regression with age as an explanatory factor). F values were calculated by always using the Type I mean squares of the next lower level term as an error term. Error df = 183.

**Table 2:** Hierarchical models ^a^ for partitioning the variation (%) in element concentrations (μg/g, d.m.) of feces of pied flycatcher (*Ficedula hypoleuca*) nestlings among study plots, broods, siblings within a brood and an individual nestling sampled two times within a day. *N* = 391 samples.

Source of Variation	df	As	Ca	Cd	Co	Cu	Ni	Pb	$\bar{X}$
Plot	5	5.7	4.0	0.0	7.9	3.1	12.4	7.7	5.8
Brood (plot)	32	27.7	5.5	13.0	25.7	15.3	24.0	12.9	17.7
Nestling (brood)	170	1.58	0.0	7.0	17.7	18.9	14.1	17.6	11.0
Time (nestling)	183	65.0	90.5	80.0	48.7	62.8	49.6	61.8	65.5
Total	390	100	100	100	100	100	100	100	

^a^ Linear models with nested structure for age-corrected values (i.e., residuals of age). The figures show relative proportions (%) of each term from total variance.

**Table 3:** Pearson correlations (r) ^a^ and repeatability coefficients ^b^ for metal levels between successive fecal samples. Repeatability is calculated both for individual and brood level.

		*n*	As	Ca	Cd	Co	Cu	Ni	Pb
Correlation	r	183	0.35	0.0074	0.16	0.36	0.35	0.35	0.32
	*p*		<0.0001	0.92	0.0028	<0.0001	<0.0001	<0.0001	<0.0001
Repeatability, individual level		366	0.36	0.0018	0.18	0.48	0.38	0.49	0.41
Repeatability, brood level		76	0.64	0.15	0.39	0.79	0.71	0.78	0.70
Repeatability, plot level		6	0.77	0.42	0.23	0.79	0.69	0.92	0.76

^a^ Correlations were calculated with log_10_-transformed values. ^b^ Intra-class correlation coefficients were calculated with log_10_-transformed values, following Lessells and Boag (1987).

**Table 4:** Geometric means (±95% confidence limits) for metal concentrations (µg/g, d.m.) in urate and feces of *F. hypoleuca* nestlings.

Matrix	Mean Mass Proportion (% ± SD)	As	Cd	Co	Cu	Ni	Pb
Urate	39 ± 17%	3.31	1.26	2.53	186	33.9	5.66
		(2.64–4.16)	(1.03–1.54)	(1.96–3.27)	(152–228)	(26.2–43.8)	(4.36–7.34)
		*n* = 39	*n* = 39	*n* = 39	*n* = 38	*n* = 39	*n* = 39
Feces	61 ± 17%	6.87	2.86	5.82	382	84.2	12.7
		(5.39–8.77)	(2.32–3.53)	(4.75–7.12)	(326–448)	(67.2–105)	(10.1–16.0)
		*n* = 40	*n* = 40	*n* = 40	*n* = 40	*n* = 40	*n* = 40
Combined sample ^a^		5.63	2.27	4.60	308	64.9	10.2
		(4.38–7.24)	(1.86–2.77)	(3.70–5.72)	(261–228)	(51.7–81.5)	(8.10–12.8)
		*n* = 39	*n* = 39	*n* = 39	*n* = 38	*n* = 39	*n* = 39
Mean (±SD) proportion (%) of metal in the fecal part ^a^		73.3 ± 15	74.5 ± 15	74.9 ± 15	73.4 ± 16	76.3 ± 15	74.7 ± 14
Proportion of variation (R^2^) explained by relative mass of the fecal part ^b^		9.3	0.0	5.6	5.4	1.0	3.2
Pearson correlation between urine and feces (*p*-value)		0.84	0.50	0.71	0.60	0.74	0.65
		*p* < 0.0001	*p* = 0.0011	*p* < 0.0001	*p* < 0.0001	*p* < 0.0001	*p* < 0.0001

^a^ Takes into account the relative mass (d.m.) proportions of urate and feces. ^b^ Linear regression.

## Supplementary Materials

The following are available online at https://www.mdpi.com/2305-6304/8/4/124/s1, Figure S1: Hierarchical clustering of 24 elements in fecal samples of the pied flycatcher (*Ficedula hypoleuca*) nestlings based on their Pearson correlation matrix., Table S1: Study plots, their distances to Harjavalta copper-nickel smelter and sample sizes for fecal samples collected from the pied flycatcher (*Ficedula hypoleuca*) nestlings., Table S2: Range and quartiles (Q25, median, Q75) for element concentrations (μg/g, d.m.) in the feces of the pied flycatcher (*Ficedula hypoleuca*) in summer 2017. Proportion of samples below the limit of quantification (LOQ). *N* = 391.

## References

[1] Dauwe T., Bervoets L., Blust R., Pinxten R., Eens M. (2000). Can Excrement and Feathers of Nestling Songbirds Be Used as Biomonitors for Heavy Metal Pollution?. Arch. Environ. Contam. Toxicol..

[2] Coeurdassier M., Fritsch C., Faivre B., Crini N., Scheifler R. (2012). Partitioning of Cd and Pb in the blood of European blackbirds (*Turdus merula*) from a smelter contaminated site and use for biomonitoring. Chemosphere.

[3] Espín S., García-Fernández A.J., Herzke D., Shore R.F., Van Hattum B., Martínez-López E., Coeurdassier M., Eulaers I., Fritsch C., Gómez-Ramírez P. (2016). Tracking pan-continental trends in environmental contamination using sentinel raptors—What types of samples should we use?. Ecotoxicology.

[4] Sadeghi M., Ghasempouri S.M., Bahramifar N. (2017). Xenobiotic and essential metals biomonitoring by feathers: Molting pattern and feather regrowth sequence in four dominant waterfowl. Int. J. Environ. Sci. Technol..

[5] Clapp J.B., Bevan R.M., Singleton I. (2012). Avian Urine: Its Potential as a Non-Invasive Biomonitor of Environmental Metal Exposure in Birds. Water Air Soil Pollut..

[6] Martínez-Haro M., Taggart M.A., Lefranc H., Martin-Doimeadios R.C., Green A.J., Mateo R. (2013). Monitoring of Pb exposure in waterfowl ten years after a mine spill through the use of noninvasive sampling. PLoS ONE.

[7] Lifshitz N., Clair C.C.S. (2016). Coloured ornamental traits could be effective and non-invasive indicators of pollution exposure for wildlife. Conserv. Physiol..

[8] Belskii E.A., Bezel V.S., Lyakhov A.G. (1995). Characteristics of the reproductive indices of birds nesting in tree hollows under conditions of technogenic pollution. Russ. J. Ecol..

[9] Janssens E., Dauwe T., Van Duyse E., Beernaert J., Pinxten R., Eens M. (2003). Effects of Heavy Metal Exposure on Aggressive Behavior in a Small Territorial Songbird. Arch. Environ. Contam. Toxicol..

[10] Costa R., Eeva T., Eira C., Vaqueiro J., Medina P., Vingada J. (2014). Great tits breeding performance and mercury contamination from the paper and pulp industry in the west coast of Portugal. Chem. Ecol..

[11] Berglund Å.M.M., Rainio M.J., Eeva T. (2015). Temporal Trends in Metal Pollution: Using Bird Excrement as Indicator. PLoS ONE.

[12] Nyholm N.E.I., Donker M., Eijsackers H., Heimback F. (1994). Heavy Metal Tissue Levels, Impact on Breeding and Nestling Development in Natural Populations of Pied Flycatchers (Aves) in the Pollution Gradient from a Smelter.

[13] Dauwe T., Janssens E., Bervoets L., Blust R., Eens M. (2004). Relationships between metal concentrations in great tit nestlings and their environment and food. Environ. Pollut..

[14] Carere C., Groothuis T.G.G., Möstl E., Daan S., Koolhaas J.M. (2003). Fecal corticosteroids in a territorial bird selected for different personalities: Daily rhythm and the response to social stress. Horm. Behav..

[15] Cook N.J. (2012). Review: Minimally invasive sampling media and the measurement of corticosteroids as biomarkers of stress in animals. Can. J. Anim. Sci..

[16] Joo S., Park S. (2012). Identification of bird species and their prey using DNA barcode on feces from Korean traditional village groves and forests (maeulsoop). Anim. Cells Syst..

[17] Rytkönen S., Vesterinen E.J., Westerduin C., Leviäkangas T., Vatka E., Mutanen M., Välimäki P., Hukkanen M., Suokas M., Orell M. (2019). From feces to data: A metabarcoding method for analyzing consumed and available prey in a bird-insect food web. Ecol. Evol..

[18] Song S.J., Sanders J.G., Delsuc F., Metcalf J., Amato K., Taylor M.W., Mazel F., Lutz H.L., Winker K., Graves G.R. (2020). Comparative Analyses of Vertebrate Gut Microbiomes Reveal Convergence between Birds and Bats. mBio.

[19] Berglund Å.M. (2018). Evaluating blood and excrement as bioindicators for metal accumulation in birds. Environ. Pollut..

[20] Nam D.-H., Lee D.-P. (2006). Monitoring for Pb and Cd pollution using feral pigeons in rural, urban, and industrial environments of Korea. Sci. Total Environ..

[21] Smith P.N., Cobb G.P., Godard-Codding C.A., Hoff D., McMurry S.T., Rainwater T.R., Reynolds K.D. (2007). Contaminant exposure in terrestrial vertebrates. Environ. Pollut..

[22] Sanderfoot O.V., Holloway T. (2017). Air pollution impacts on avian species via inhalation exposure and associated outcomes. Environ. Res. Lett..

[23] Peakall D., Burger J. (2003). Methodologies for assessing exposure to metals: Speciation, bioavailability of metals, and ecological host factors. Ecotoxicol. Environ. Saf..

[24] Bervoets L., Snoeijs T., Dauwe T., Eens M., Blust R. (2006). Calcium availability influences lead accumulation in a passerine bird. Anim. Biol..

[25] Elder A., Nordberg G.F., Kleinman M. (2015). Chapter 3—Routes of Exposure, Dose, and Toxicokinetics of Metals, Handbook on the Toxicology of Metals.

[26] Berglund Å.M.M., Koivula M., Eeva T. (2011). Species- and age-related variation in metal exposure and accumulation of two passerine bird species. Environ. Pollut..

[27] Nordberg G., Fowler B.A., Nordberg M. (2015). Handbook on the Toxicology of Metals.

[28] Kalisínska E. (2019). Mammals and Birds as Bioindicators of Trace Element Contaminations in Terrestrial Environments: An Ecotoxicological Assessment of the Northern Hemisphere.

[29] Davis R.E. (1927). The nitrogenous constituents of hen urine. J. Biol. Chem..

[30] Crouch N.M.A., Lynch V.M., Clarke J.A. (2019). A re-evaluation of the chemical composition of avian urinary excreta. J. Ornithol..

[31] Casotti G., Braun E.J. (2004). Protein location and elemental composition of urine spheres in different avian species. J. Exp. Zool..

[32] Ishihara N., Matsushiro T. (1986). Biliary and Urinary Excretion of Metals in Humans. Arch. Environ. Health Int. J..

[33] Belskii E.A., Bezel V.S., Polents E.A. (1995). Early stages of the nesting period of hollow-nesting birds under conditions of industrial pollution. Russ. J. Ecol..

[34] Costa R., Eeva T., Eira C., Vaqueiro J., Vingada J. (2012). Trace Elements in Faeces of Great Tit Nestlings in Relation to Breeding Performance in Coastal Areas in Central Portugal. Arch. Environ. Contam. Toxicol..

[35] Kiikkilä O. (2003). Heavy-metal pollution and remediation of forest soil around the Harjavalta Cu-Ni smelter, in SW Finland. Silva Fenn..

[36] Kozlov M.G., Zvereva E., Zverev V. (2009). Impacts of Point Polluters on Terrestrial Biota.

[37] Eeva T., Holmström H., Espín S., Sánchez-Virosta P., Klemola T. (2018). Leaves, berries and herbivorous larvae of bilberry *Vaccinium myrtillus* as sources of metals in food chains at a Cu-Ni smelter site. Chemosphere.

[38] Eeva T., Espín S., Ruiz S., Sánchez-Virosta P., Koivula M.J. (2017). Polluted environment does not speed up age-related change in reproductive performance of the Pied Flycatcher. J. Ornithol..

[39] Berglund Å.M.M., Koivula M.J., Eeva T. (2012). Decreased metal accumulation in passerines as a result of reduced emissions. Environ. Toxicol. Chem..

[40] Lambrechts M.M., Adriaensen F., Ardia D.R., Artemyev A.V., Atiénzar F., Bańbura J., Barba E., Bouvier J.-C., Camprodon J., Cooper C.B. (2010). The Design of Artificial Nestboxes for the Study of Secondary Hole-Nesting Birds: A Review of Methodological Inconsistencies and Potential Biases. Acta Ornithol..

[41] Eeva T., Lehikoinen E., Sunell C. (1997). The quality of pied flycatcher (*Ficedula hypoleuca*) and great tit (*Parus major*) females in an air pollution gradient. Ann. Zool. Fenn..

[42] Cramp S., Perrins C.M. (1993). The Birds of the Western Palearctic VII.

[43] Eeva T., Ryömä M., Riihimäki J. (2005). Pollution-related changes in diets of two insectivorous passerines. Oecologia.

[44] SAS Institute Inc (2013). Base SAS 9.4 Procedures Guide: Statistical Procedures.

[45] EPA (2000). Guidance for Data Quality Assessment: Practical Methods for Data Analysis.

[46] Sánchez-Virosta P., Espín S., Ruiz S., Stauffer J., Kanerva M., García-Fernández A.J., Eeva T. (2019). Effects of calcium supplementation on oxidative status and oxidative damage in great tit nestlings inhabiting a metal-polluted area. Environ. Res..

[47] Saulnier A., Bleu J., Boos A., El Masoudi I., Ronot P., Zahn S., Del Nero M., Massemin S. (2020). Consequences of trace metal cocktail exposure in zebra finch (*Taeniopygia guttata*) and effect of calcium supplementation. Ecotoxicol. Environ. Saf..

[48] Lessells C.M., Boag P.T. (1987). Unrepeatable Repeatabilities: A Common Mistake. Auk.

[49] Harper D.G. (1994). Some comments on the repeatability of measurements. Ringing Migr..

[50] Afik D., Karasov W.H. (1995). The Trade-Offs Between Digestion Rate and Efficiency in Warblers and Their Ecological Implications. Ecology.

[51] Michalski M., Nadolski J., Marciniak B., Loga B., Banbura J. (2011). Faecal analysis as a method of nestling diet determination in insectivorous birds: A case study in Blue Tits *Cyanistes caeruleus* and Great Tits *Parus major*. Acta Ornithol..

[52] Belskii E., Belskaya E. (2013). Diet composition as a cause of different contaminant exposure in two sympatric passerines in the Middle Urals, Russia. Ecotoxicol. Environ. Saf..

[53] Graveland J. (1994). Decline of snail abundance on acidified soils causes poor reproduction in forest passerines. J. Ornithol..

[54] Eeva T., Rainio K., Suominen O. (2010). Effects of pollution on land snail abundance, size and diversity as resources for pied flycatcher, *Ficedula hypoleuca*. Sci. Total Environ..

[55] Louis V.L.S., Breebaart L. (1991). Calcium Supplements in the Diet of Nestling Tree Swallows near Acid Sensitive Lakes. Condor.

[56] Wilkin T.A., Gosler A.G., Garant D., Reynolds S.J., Sheldon B.C. (2009). Calcium effects on life-history traits in a wild population of the great tit (*Parus major*): Analysis of long-term data at several spatial scales. Oecologia.

[57] Pounds J.G. (1984). Effect of lead intoxication on calcium homeostasis and calcium-mediated cell function: A review. NeuroToxicology.

[58] Eeva T., Lehikoinen E. (2004). Rich calcium availability diminishes heavy metal toxicity in Pied Flycatcher. Funct. Ecol..

[59] Migula P., Glowacka E., Nuorteva S.-L., Nuorteva P., Tulisalo E. (1997). Time-related effects of intoxication with cadmium and mercury in the red wood ant. Ecotoxicology.

[60] Silverin B., Andersson G. (1984). Föda hos svartvita flugsnappare *Ficedula hypoleuca*: En jämförelse mellan vuxna fåglar och boungar. Vår Fågelvärld.

[61] Schmitz A. (2016). Respiration in spiders (Araneae). J. Comp. Physiol. B.

[62] Custer C.M., Yang C., Crock J.G., Shearn-Bochsler V., Smith K.S., Hageman P.L. (2008). Exposure of insects and insectivorous birds to metals and other elements from abandoned mine tailings in three Summit County drainages, Colorado. Environ. Monit. Assess..

[63] Wasserman R.H. (1998). Strontium as a Tracer for Calcium in Biological and Clinical Research. Clin. Chem..

[64] Blum J.D., Taliaferro E.H., Holmes R.T. (2001). Determining the sources of calcium for migratory songbirds using stable strontium isotopes. Oecologia.

[65] Qiu R., Qiu R., Tang Y., Wang S. (2014). Cadmium–zinc exchange and their binary relationship in the structure of Zn-related proteins: A mini review. Metallomics.

[66] Boyer J.L. (2013). Bile Formation and Secretion. Compr. Physiol..

[67] Gregus Z. (1986). Disposition of metals in rats: A comparative study of fecal, urinary, and biliary excretion and tissue distribution of eighteen metals. Toxicol. Appl. Pharmacol..

[68] Csanaky I., Gregus Z. (2002). Species variations in the biliary and urinary excretion of arsenate, arsenite and their metabolites. Comp. Biochem. Physiol. Part C Toxicol. Pharmacol..

[69] Pain D.J., Mateo R., Green R.E. (2019). Effects of lead from ammunition on birds and other wildlife: A review and update. Ambio.

