# Supplementary material for: Bird Feces as Indicators of Metal Pollution: Pitfalls and Solutions
Source: Toxics. 2020 Dec 18;8(4):124. doi: 10.3390/toxics8040124 (PMC7767019; doi:10.3390/toxics8040124)
Supplement: Supplementary file 1 [file toxics-08-00124-s001.pdf]

Supplementary materials to: Eeva, T., Raivikko, N., Espín, S., Sánchez-Virosta, P., Ruuskanen, S., Sorvari, J., Rainio, M. (2020): Bird feces as indicators of metal pollution: pitfalls and solutions.

Table S1. Study plots, their distances to Harjavalta copper-nickel smelter[1] and sample sizes for fecal samples collected from the pied flycatcher (*Ficedula hypoleuca*) nestlings.

Sampling year	Plot ID	Distance to smelter (km)	Number of broods sampled	Total number of nestlings	Number of sampled nestlings (1st sample)[2]	Number of sampled nestlings (2nd sample)[2]	Total number of samples
2017	01	1.14	10	55	51	51	102
	02	0.31	4	19	19	18	37
	03	1.74	7	42	42	39	81
	21	0.75	3	18	17	17	34
	22	0.86	4	18	17	17	34
	25	1.95	10	56	55	53	108
	Total		38	208	201	195	396
2018	01	1.14	6	35	12	N.A.	12
	02	0.31	4	20	8	N.A.	8
	03	1.74	3	20	6	N.A.	6
	21	0.75	2	11	4	N.A.	4
	22	0.86	1	7	2	N.A.	2
	25	1.95	4	25	8	N.A.	8
	Total		20	118	40		40

[1] For a map of the study area and location of study plots see: T Eeva, H Holmström, S Espín, P Sánchez-Virosta, T Klemola (2018). Leaves, berries and herbivorous larvae of bilberry *Vaccinium myrtillus* as sources of metals in food chains at a Cu-Ni smelter site. Chemosphere 210: 859-866.

[2] In 2017, all nestlings in a brood were sampled and two samples from the same nestlings were collected during the same day when possible. In 2018, two nestlings per brood were sampled once and samples were further split in two parts: feces (n = 40) and urate (n = 40).

Table S2. Range and quartiles (Q25, median, Q75) for element concentrations (µg/g, d.m.) in the feces of the pied flycatcher (*Ficedula hypoleuca*) in summer 2017. Proportion of samples below the limit of quantification (LOQ). N = 391.

Element	*Group*[a]	*Range (min – max)*	*Q25*	*Median*	*Q75*	*%<LOQ*
Aluminum (Al)	2	1.48 - 31800	175	418	1460	0
Arsenic (As)	2	<LOQ - 168	2.15	5.59	12.3	12.1
Boron (B)	1	<LOQ - 89.3	5.15	7.75	11.2	2.80
Cadmium (Cd)	2	0.205 - 62.9	3.53	5.94	8.59	0
Calcium (Ca)	1	60.8 - 264000	569	1280	7030	0
Chromium (Cr)	2	<LOQ - 106	1.21	2.69	6.52	1.52
Cobalt (Co)	1,2	0.207 - 135	1.63	3.32	5.87	0
Copper (Cu)	1,2	16.2 - 8060	160	239	364	0
Iron (Fe)	1	34.8 - 26300	432	890	2290	0
Lead (Pb)	2	0.212 - 196	2.98	5.85	10.5	0
Lithium (Li)	3	6.66 - 496	35.8	61.9	87.2	0
Magnesium (Mg)	1	12.8 - 21200	2210	2870	3710	0
Manganese (Mn)	2	7.69 - 1660	116	165	246	0
Molybdenum (Mo)	1	0.161 - 44.7	1.52	2.41	3.85	0
Nickel (Ni)	2	4.22 - 912	27.0	48.1	90.4	0
Phosphorus (P)	1	528 - 162000	8200	10900	13400	0
Potassium (K)	1	233 - 153000	9850	13500	18200	0
Rubidium (Rb)	3	0.83 - 107	5.46	7.91	11.6	0
Silicon (Si)	1	28.5 - 5390	306	481	793	0
Sodium (Na)	1	52.3 - 55200	2200	3700	5940	0
Strontium (Sr)	3	0.109 - 1160	6.50	10.7	25.5	0
Sulphur (S)	1	421 - 54500	4740	5860	6920	0
Titanium (Ti)	3	0.893 - 1050	14.2	33.6	120	0
Zinc (Zn)	1,2	14.1 - 3610	314	433	584	0

[a] Element categories: 1 = Essential trace elements, 2 = ATSDR's list toxic elements, 3 = Other minor elements.

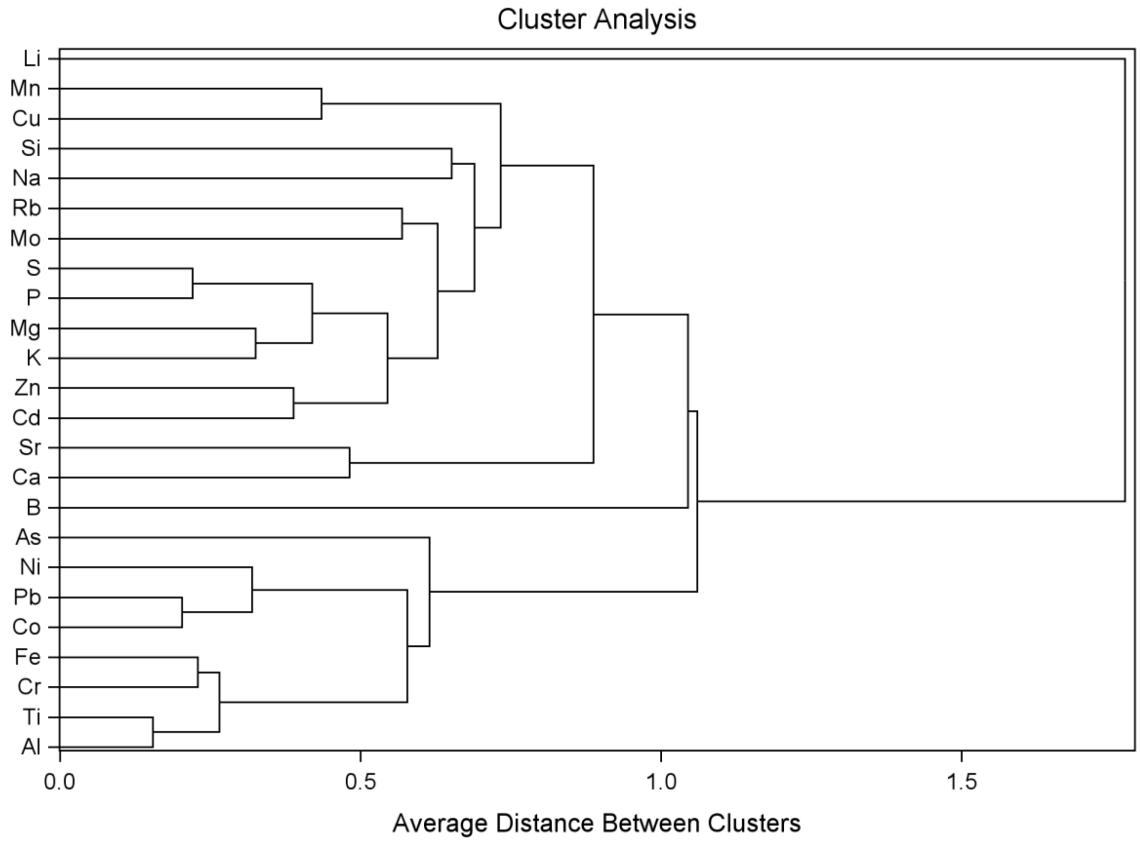

Figure S1. Hierarchical clustering of 24 elements in fecal samples of the pied flycatcher (*Ficedula hypoleuca*) nestlings based on their Pearson correlation matrix. N = 391 for each element.
